# Fear of disease progression in carriers of the m.3243A > G mutation

**DOI:** 10.1186/s13023-018-0951-y

**Published:** 2018-11-13

**Authors:** José A. E. Custers, Paul de Laat, Saskia Koene, Jan Smeitink, Mirian C. H. Janssen, Christianne Verhaak

**Affiliations:** 1 Department of Medical Psychology, Radboud Center for Mitochondrial Medicine, Radboud University Medical Center, Geert Grooteplein Zuid 10, PO Box 9101, 6500 HB Nijmegen, The Netherlands; 2 Department of Pediatrics, Radboud Center for Mitochondrial Medicine, Radboud University Medical center, Geert Grooteplein Zuid 10, PO Box 9101, 6500 HB Nijmegen, The Netherlands; 3 Department of Internal Medicine, Radboud Center for Mitochondrial Medicine, Radboud University Medical Center, Geert Grooteplein Zuid 10, PO Box 9101, 6500 HB Nijmegen, the Netherlands

**Keywords:** Mitochondrial disease, M.3243A > G mutation, Fear of progression, Quality of life, Mental health

## Abstract

**Background:**

Being diagnosed with mitochondrial disease due to the m.3243A > G mutation is frequently preceded by a long diagnostic process. The disease itself is characterized by heterogeneous course and expression, so leaving patients with considerable uncertainty regarding their prognosis and treatment possibilities. This could easily result in fear of disease progression. This study investigated the presence of this fear and its correlates with genetic characteristics and clinical disease severity in m.3243A > G carriers.

**Methods:**

In total 125 eligible m.3243A > G mutation carriers were invited to participate in this cross-sectional study. After informed consent, participants completed questionnaires including items on socio-demographics, fear of progression, depression, anxiety, and quality of life. Clinical disease severity was assessed by the NMDAS questionnaire. Heteroplasmy levels were assessed in leucocytes, urine epithelial cells and buccal mucosa.

**Results:**

Seventy-six carriers participated in this study. Results showed that 18% reported high fear of progression. Fear of progression was significantly related to all domains of quality of life. Furthermore, fear of progression was moderately correlated with feelings of depression (*r* = .37), and anxiety (*r* = .44). Patients with moderate or severe clinical symptoms on the NMDAS experienced more fear of progression than patients with mild clinical symptoms. Fear of progression was weakly correlated with heteroplasmy in leucocytes (*r* = .27) and buccal mucosa (*r* = .31).

**Conclusions:**

A substantial part of m.3243A > G mutation carriers experience high levels of fear of progression which coincide with significantly lower quality of life. Only a small relation with disease characteristics was found. The impact of receiving a diagnosis without therapeutic possibilities on fear is important to consider.

## Background

Fear of disease progression (FoP) is amongst the most important stressors for patients with a life-threatening disease [1]. Mitochondrial diseases are rare multi-system diseases that result from problems due to disturbed mitochondrial oxidative phosphorylation, the final pathway in the production of ATP, caused by mutations in either the nuclear or mitochondrial DNA [2–4]. Failure of the mitochondria to perform normally results a.o. in decreased energy production, cell injury or cell death, and eventual organ dysfunction or failure [5]. Although new pharmacological interventions are being explored, currently no cure or substantially alleviating therapy is available for these disorders and care is focused on alleviating the broad range of symptoms of the disease [6].

Many different genetic defects of mtDNA exist which, in general, may cause a specific clinical syndrome. However, the relationship between genotype and phenotype is not absolute and syndromes might overlap [7]. The life of the individual with mitochondrial disease can be greatly compromised, according to the degree and nature of mitochondrial malfunction. The serious nature and erratic, complex presentation of mitochondrial disease often make it difficult to establish a diagnosis and may contribute to increased stress and worry among patients and their relatives.

Fear of disease progression has been defined as a reactive, consciously perceived fear that develops from a serious, potential life-threatening or disabling disease or its treatment [1]. Since a mitochondrial disease has serious consequences in most patients, they perceive disease progression as a realistic threat. In this respect, FoP does not belong to the group of anxiety disorders for which an irrational fear is usually one of the key characteristics. Basically, FoP is an appropriate response to the real threats of diagnosis, treatments and course of illness. However, the level of FoP can range between functional and dysfunctional ends. Elevated levels of fear of progression that become dysfunctional, affecting quality of life, are in need for treatment [8]. Previous studies revealed that FoP was evident in other patients with cancer, rheumatoid arthritis, diabetes mellitus, Parkinson’s disease, Crohn’s disease, and multiple sclerosis [1, 9]. However, information on the impact of a rare disease without therapeutic possibilities, a very heterogeneous course and a hereditary character on FoP is scarce.

The aim of the present study is to investigate the prevalence of fear of progression in patients with a mitochondrial disease. In addition, demographical, medical and psychological correlates of fear of progression are assessed. We focused on patients with a clinically and genetically heterogeneous phenotype known as the MELAS spectrum disorders, most commonly caused by the m.3243 A > G mutation in mitochondrial DNA. This spectrum is characterized by decreased exercise tolerance, elevated lactic acid levels, cardiac abnormalities, diabetes, and neurological symptoms like migraine, psychiatric problems, seizures, ataxia, myopathy, impaired vision or hearing, or disturbances in consciousness [10].

## Methods

### Participants

Patients with a mitochondrial disease due to the m.3243 A > G mutation of the Radboud Center for mitochondrial medicine, were invited to participate in this study. Participants had to be able to read and write in Dutch.

### Procedure

This cross-sectional study was part of a longitudinal observational cohort study on patient reported outcomes approved (NL32683.091.10) by the local ethical committee of the Radboud university medical center, Nijmegen, the Netherlands. Patients were invited to participate in the study by their treating physician. After given informed consent, patients received an email with a link to a private, secure website that presented a set of questionnaires on demographic variables and psychological factors that could be administered at home at time of first access or later. Patients were asked to complete the total set of questionnaires within 1 week. The Newcastle Mitochondrial Disease Assessment Scale (NMDAS) [11] was assessed during clinical investigation. Questionnaires regarding FoP were added in the last phase of the longitudinal study (two to five years after baseline) and analyzed for this study.

### Instruments

*Genotype* was assessed in terms of heteroplasmy levels of the m.3243A > G mutation in leucocytes, urine epithelial cells and buccal mucosa.

*Disease Manifestation* was assessed by the Newcastle Mitochondrial Disease Scale [11]. The NMDAS is a validated method to monitor the clinical expression of mitochondrial disease and to follow-up the course of the disease in time. It consists of the following three sections: (1) Current functioning: general functioning of the patient in the past 4 weeks (2) System specific involvement, to gain insight in the functioning of individual organ-systems, (3) Current clinical assessment, a general and neurological clinical examination, gives insight in the current clinical functional status of the patient. Section 1 to 3 of the NMDAS consists respectively of ten, nine and ten questions, which can be scored from 0 (no involvement) to 5 (severe involvement). A NMDAS score of 0 indicates no clinical manifestation, NMDAS scores of 1 to 5 were defined as mild clinical manifestation, scores 6 to 20 as moderate and scores above 20 as severe clinical manifestation [10].

*Fear of Progression* was assessed with the Dutch translation of the FoP-Q-SF [12–14]. The FoP-Q-SF consists of 12 items pertaining to four dimensions (affective reactions, partnership/family issues, occupation and loss of autonomy) that are scored from 1 (never) to 5 (very often), where higher scores indicate more FoP. A cut-off score of ≥34 was used to define high FoP [15, 16].

*Quality of life* was assessed with the RAND-SF36. The RAND-SF36 assesses several dimensions of quality of life (physical functioning, social functioning, emotional functioning, general health status, perceived change in health status, fatigue and pain). Scores on different scales range from 0 (maximum limitations) to 100 (optimal functioning) [17, 18].

*Mental functioning* was assessed with the Hospital Anxiety and Depression Scale (HADS). This questionnaire includes 14 items divided into two subscales (Depression and Anxiety), each with seven items. Higher scores indicate more anxiety, depression, and psychological distress. The HADS does not contain any somatic items that could be confounded with symptoms associated with a physical illness. A total score of 11 or higher indicates high distress [19, 20].

### Data-analyses

Data analysis was performed using SPSS version 22.0. Descriptive statistics were used to describe the characteristics of the sample as well as patient reported outcomes on FoP, quality of life, and mental functioning. Correlational analyses identified the main correlates of FoP with demographical, disease-related and psychological variables. To categorize the results, Cohen’s effect size categories were used: small (|r| ≤ 0.3), moderate (0.3 < |r| < 0.5), or large (|r| ≥ 0.5) [21].

## Results

### Sample characteristics

Of 125 eligible patients with the m.3243 A > G mutation, 76 patients completed the questionnaires, a response rate of 61%. Demographic characteristics are displayed in Table 1. Differences between response group and non-responders were assessed on demographic and clinical characteristics. There were no differences between responders and non-responders with regard to age (*t*(122) = −.77, *p* = .45) or gender (*Χ*^*2*^(1) = 3.17, *p* = .08). For clinical characteristics, the groups did not differ on levels of heteroplasmy, NMDAS subscales or total NMDAS score.

**Table 1:** Sample characteristics (*n* = 76)

	Mean	Median	Range
Age (years)	47.3	47.6	15–69
Heteroplasmy level			
Leucocytes	19.9	17.0	0–56
Urine epithelial cells	51.0	50.0	5–96
Buccal mucosa	34.4	34.5	2–63
NMDAS			
1 current functioning	8.1	6.0	0–43
2 system specific involvement	6.5	6.0	0–30
3 current clinical assessment	3.3	1.0	0–33
Total NMDAS	17.7	16.0	0–86
	N	%	
Gender: male	20	26%	
Marital status			
Married/partnership	62	82%	
Children: yes	55	72%	
Educational level			
Primary	6	8%	
Secondary	46	61%	
Tertiary	24	31%	
Employment status: paid work	36	47%	

### Genotype

Levels of heteroplasmy were assessed in leucocytes (*M* = 19,9%; *SD* = 12.9; range 0–56), urinary epithelial cells (*M* = 51.0%; *SD* = 25.1; range 5–96) and buccal mucosa (*M* = 34.4%; *SD* = 14.8; range 2–63).

### Disease manifestation

Mean total NMDAS score was 17.7 (SD = 15.5) varying from 0 to 86. Of the total sample, three patients were asymptomatic (“dormant carrier” of the mutation), 17% of the patients showed mild symptoms, 45% moderate and 33% had severe symptoms of mitochondrial disease.

### Relationship genotype and disease manifestation

Significant correlations were found between NMDAS total scores and levels of heteroplasmy in leucocytes (*r* = .275, *p* = .03), levels of heteroplasmy in urinary epithelial cells (*r* = .270, *p* = .03), and levels of heteroplasmy in buccal mucosa (*r* = .438, *p* = .001). Furthermore, a significant correlation was found between clinical severity (as indicated by mild, moderate or severe) and levels of heteroplasmy in buccal mucosa (*r* = .475, *p <* .001). No significant correlations were found between clinical severity and levels of heteroplasmy in leucocytes or urinary epithelial cells.

### Fear of progression

Cronbach’s alpha for the FoP-Q-SF in this sample was 0.84. The FoP mean score (FoP-Q-SF) was 26.2 (SD = 7.7; range 12–41). By applying the cutoff score ≥ 34, 14 patients (18.4%) were classified as having high FoP. Means and standard deviations for the individual items of the FoP-Q-SF are displayed in Table 2. Highest mean scores were related to the possibility that children can inherit the disease, progression of the disease and worrying about family when something would happen with the patient. Lowest mean scores were related to medical appointments or treatments.

**Table 2:** Mean scores of individual FoP-Q-SF items

Item	Mean^a^	SD
1. Being afraid of disease progression	2.58	0.93
2. Being nervous prior to doctor’s appointments or periodic examinations	1.87	0.94
3. Being afraid of pain	2.03	0.98
4. Being afraid of becoming less productive at work	2.09	1.10
5. Having physical symptoms (e.g., rapid heartbeat, stomache ache)	2.13	1.04
6. Being afraid of the possibility that the children could contract the disease	2.63	1.51
7. Being afraid of relying on strangers for activities of daily living	2.24	1.04
8. Being afraid of no longer being able to pursue hobbies	2.39	1.03
9. Being afraid of severe medical treatments in course of illness	1.88	0.85
10. Worrying that medications could damage the body	1.84	0.94
11. Worrying what will become of family if something happens to me	2.51	1.13
12. Being afraid of not being able to work anymore	1.99	1.13

^a^On a 5-point Likert scale, where 1 = never and 5 = very often

### Quality of life

Table 3 shows the mean (±SD) scores for the quality of life domains as assessed by the RAND-36. Scores on the subscales of the RAND-36 ranged from 44.2 to 73.2, indicating a moderate quality of life. Patients reported general health status, perceived change in health status and fatigue to be significant problems.

**Table 3:** Quality of life (RAND-36) scores

	Mean	SD
Physical functioning	69.1	24.7
Social functioning	69.9	22.7
Emotional functioning	72.9	16.6
General health status	44.2	23.8
Perceived change in health status	44.7	22.5
Pain	73.2	26.0
Fatigue	56.6	21.3

### Mental functioning

Mean scores for the HADS total score (*M* = 10.3, *SD* = 6.8) and subscales anxiety (*M* = 5.2, *SD* = 3.5) and depression (*M* = 5.1, *SD* = 4.1) were calculated. Results indicated that 25 and 26.3% of the patients indicated clinical relevant symptoms of respectively anxiety and depression. By applying the cut-off score of ≥11 on the total score, 46% of the patients experienced clinical relevant levels of general distress.

### Relationship fear of progression and demographical, medical and psychosocial variables

#### Fear of progression and demographics

Fear of progression was not related to age (*r* = −.07, *p* = .548; *t*(74) = .20, *p* = .84). A marginally significant relationship (*t*(74) = − 1.9, *p* = .06) was found between gender and FoP indicating that women (*M* = 27.2, *SD* = 7.9) tended to experience more FoP compared to men (*M* = 23.4, *SD* = 6.7). No significant relationships were found between FoP and marital status (*p* = .20), having children (*p* = .51), level of education (*p* = .53) or employment status (*p* = .36).

#### Fear of progression and medical related variables

No significant correlations were found between FoP and NMDAS scores. To assess the extend to what FoP was related to the degree of disease manifestation a one-way ANOVA was performed with three NMDAS categories (mild, moderate, severe) and FoP as dependent variable. Results indicated that scores on FoP differed significantly between NMDAS severity groups (*F*(2,71) = 3.59, *p* = .03). Patients in the moderate (M = 27.4; SD = 8.4) and severe (M = 27.6; SD = 6.6) group experienced significantly more FoP than patients in the mild group (M = 21.3; SD = 6.6).

With regard to genotype, a small but significant relationship was found between FoP and levels of heteroplasmy in leucocytes (*r* = .28, *p* = .03) and a moderate but significant relationship between FoP and levels of heteroplasmy in buccal mucosa (*r* = .31, *p* = .02). No significant relationship was found between FoP and levels of heteroplasmy in urine epithelial cells (*p* = .64).

#### Fear of progression and psychosocial variables

With regard to quality of life, moderate significant correlations were found between FoP and physical functioning (*r* = −.47, *p* < .001), social functioning (*r* = −.39, *p* < .001), general health status (*r* = −.48, *p* < .001), pain (*r* = −.40, *p* < .001) and fatigue (*r* = −.44, *p* < .001). Small relationships were found between FoP and perceived change in health status (*r* = −.27, *p* = .02) and emotional functioning (*r* = −.29, *p* = .01).

For mental functioning, FoP was moderately correlated with depression (*r* = .37, *p* = .001), anxiety (*r* = .44, *p* < .001), and general distress (*r* = .45, *p* < .001).

## Discussion

This is the first exploratory study to investigate fear of progression and one of the few studies reporting on quality of life in patients with a MELAS spectrum disorder. About one-fifth (18.4%) of the patients showed elevated levels of FoP and 46% reported clinical levels of general distress. Furthermore, patients reported a moderate quality of life with problems reported on general health status and fatigue. FoP was moderately related to components of quality of life, mental health and medical variables. Furthermore, higher levels of heteroplasmy were small but significantly correlated with more disease manifestation. It was hypothesized that FoP would be high in patients with a MELAS spectrum disorder given its uncertain and unpredictable disease course, premature knowledge about its character and absence of treatment possibilities. Compared to other studies using the FoP-Q-SF the percentage of high FoP found in this study was lower (e.g. hematological cancer 23–26%) [15, 16]. Furthermore the mean FoP score of patients with a MELAS spectrum disorder was significant lower than patients with systemic sclerosis [14] and patients with breast cancer [22]. Inspection of the single items of the FoP-Q-SF revealed that carriers were most afraid of the possibility that their children could inherit the disease, progression of the disease and worrying about their family if something happens to them. These results confirm that dealing with this mitochondrial disease is characterized by dealing with uncertainty about future, disease course and hereditary concerns. This is different for other groups whereas cancer patients predominantly fear dying and the unpredictability of progression. Patients with chronic arthritis fear being physically dependent on others and the most common anxiety of patients with diabetes are long-term complications [1]. Insight in why patients react differently to the news that they have a mitochondrial disease and why some are more fearful than others for disease progression could be provided by Leventhal’s Self Regulation Model of Illness [23]. External and internal stimuli generate a subjective perception of a health threat and concomitant emotions (e.g. fear/distress), leading to coping strategies and appraisal of health outcomes. Internal stimuli might be interpreted as reminders of threat that the disease progresses. This study demonstrates that patients with moderate and severe clinical symptoms as assessed by the NMDAS reported higher FoP indicating that the presence of symptoms is associated with higher fear. External triggers such as medical appointments could also increase worry about disease status and disease course. However, in this study patients reported low fear scores on the item of the FoP-Q-SF whether they are nervous prior to doctor’s appointments or periodic examinations. Given the fact that the Radboud Center for Mitochondrial Medicine is the expert center for mitochondrial disorders in the Netherlands, patients might feel reassured by the expertise and well arranged care in this center, and therewith this could be a buffer for excessive fear of progression.

Although the percentage of FoP was not as high as hypothesized, a considerable percentage of patients (46%) experienced clinical levels of general distress defined as a complex, unpleasant emotional experience characterized by comprised psychological, social and/or spiritual wellbeing [24]. Given that both general distress and FoP are associated with a lower quality of life and restrictions or problems in daily life, it is important in this patient group to assess mental functioning in clinical consultations and assist patients in accessing appropriate and available support.

A limitation of this study is its cross-sectional design. Longitudinal research could elaborate the role of FoP as mediator between medical history and personal characteristics as antecedents and functioning and quality of life as consequences. Furthermore, given the uncertain and unpredictable character of the disease a valid and reliable measurement for uncertainty could have shed light on this issue in this research. For further research it would be interesting to measure uncertainty and its correlates. With regard to measurement issues another limitation is the use of the cut-off score of the FoP-Q-SF, validated in a sample of hematological cancers. Future research should replicate the validation of this cut-off score in a sample of patients with a mitochondrial disorder. Therewith both research and clinical practice could contribute to support these patients in dealing with their disease.

## Conclusions

In conclusion, a substantial percentage of patients with a MELAS spectrum disorder reported considerable levels of FoP and distress coinciding with impairments in their quality of life. Therefore, it is important to integrate assessments of mental functioning in clinical consultations in order to assist patients in accessing appropriate and available support.

Based on the findings of this study, further research should elaborate on the concept of uncertainty about the future and should replicate and validate the cut-off score for FoP in a more heterogeneous sample of patients with diverse mitochondrial disorders. Furthermore, with regard to hereditary concerns, it might be interesting to investigate clusters of families affected by a mitochondrial disease and the influence of having family with the same progressive disease on fear of progression and uncertainty for the future.

## Abbreviations

ANOVA: Analysis of variance
FoP: Fear of progression
HADS: Hospital Anxiety and Depression Scale
MELAS: Mitochondrial encephalomyopathy lactic acidosis and stroke like episodes
NMDAS: Newcastle Mitochondrial Diseases Adult Scale
SD: Standard deviation
